# Written Advice Given by African American Smokers to Their Peers: Qualitative Study of Motivational Messages

**DOI:** 10.2196/21481

**Published:** 2021-04-30

**Authors:** Catherine S Nagawa, Jamie M Faro, Anitha J Menon, Mayuko Ito Fukunaga, Jessica H Williams, Dalton Mourao, Oluwabunmi M Emidio, Maryann Davis, Lori Pbert, Sarah L Cutrona, Thomas K Houston, Rajani S Sadasivam

**Affiliations:** 1 Department of Population and Quantitative Health Sciences University of Massachusetts Medical School Worcester, MA United States; 2 Department of Psychology University of Zambia Lusaka Zambia; 3 Department of Medicine University of Massachusetts Medical School Worcester, MA United States; 4 Meyers Primary Care Institute Worcester, MA United States; 5 University of Alabama at Birmingham Birmingham, AL United States; 6 Department of Psychiatry University of Massachusetts Medical School Worcester, MA United States; 7 Center for Healthcare Organization and Implementation Research VA Bedford Healthcare System Bedford, MA United States; 8 Section of General Internal Medicine Department of Internal Medicine Wake Forest University School of Medicine Winston-Salem, NC United States

**Keywords:** tobacco disparities, peer-to-peer, communication, smoking, cessation, thematic analysis, intervention, African American

## Abstract

**Background:**

Although African Americans have the lowest rates of smoking onset and progression to daily smoking, they are less likely to achieve long-term cessation. Interventions tailored to promote use of cessation resources in African American individuals who smoke are needed. In our past work, we demonstrated the effectiveness of a technology-assisted peer-written message intervention for increasing smoking cessation in non-Hispanic White smokers. In this formative study, we have adapted this intervention to be specific for African American smokers.

**Objective:**

We aimed to report on the qualitative analysis of messages written by African American current and former smokers for their peers in response to hypothetical scenarios of smokers facing cessation challenges.

**Methods:**

We recruited African American adult current and former smokers (n=41) via ResearchMatch between April 2017 and November 2017. We asked participants to write motivational messages for their peers in response to smoking-related hypothetical scenarios. We also collected data on sociodemographic factors and smoking characteristics. Thematic analysis was conducted to identify cessation strategies suggested by the study participants.

**Results:**

Among the study participants, 60% (25/41) were female. Additionally, more than half (23/41, 56%) were thinking about quitting, 29% (12/41) had set a quit date, and 27% (11/41) had used electronic cigarettes in the past 30 days. Themes derived from the qualitative analysis of peer-written messages were (1) behavioral strategies, (2) seeking help, (3) improvements in quality of life, (4) attitudes and expectations, and (5) mindfulness/religious or spiritual practices. Under the behavioral strategies theme, distraction strategies were the most frequently suggested strategies (referenced 84 times in the 318 messages), followed by use of evidence-based treatments/cessation strategies. Within the seeking help theme, subthemes included seeking help or support from family/friends or close social networks (referenced 56 times) and health care professionals (referenced 22 times). The most frequent subthemes that emerged from improvements in the quality of life theme included improving one’s health (referenced 22 times) and quality of life (referenced 21 times). Subthemes that emerged from the attitude and expectations theme included practicing positive self-talk (referenced 27 times), autonomy/independence from the smoking habit (referenced six times), and financial cost of smoking (referenced five times). The two subthemes that emerged from the mindfulness/religious or spiritual practices theme were use of self-awareness techniques (referenced 36 times) and religious or spiritual practices to cope (referenced 13 times).

**Conclusions:**

Our approach to adapt a prior peer-message intervention to African American smokers yielded a set of evidence-based messages that may be suitable for smokers at all phases of motivation to quit (ready to quit or not ready to quit). In future research, we plan to assess the impact of texting these messages to African American smokers in a smoking cessation trial.

## Introduction

Smoking is the leading cause of preventable death in the United States [1]. Smoking cessation programs have reduced overall smoking rates, but their impacts have not been uniformly beneficial [2,3]. Smoking-related disparities have noticeably expanded, resulting in calls for targeted cessation programs to meet the needs of minority and disadvantaged populations [3]. African Americans are disproportionately affected by tobacco-related diseases in terms of mortality and morbidity. African Americans are 20% more likely to die from heart disease than non-Hispanic Whites and have a higher incidence of lung cancer compared with the general population [4-7].

Although African Americans have the lowest rates of smoking onset, have slower progression to daily smoking [8], and report attempting to quit (63%) more often than non-Hispanic White smokers (53%) [9], they are less likely to achieve long-term cessation [10]. Tailored cessation interventions are needed to support African American smokers [11].

We conducted formative research to adapt a technology-assisted peer message intervention for African American smokers. The effectiveness of a peer-written message intervention has been demonstrated in a large randomized trial conducted among mostly non-Hispanic White smokers, who were recruited from medical practices across the United States [12,13]. In this study, participants were enrolled into a web-assisted tobacco intervention. The intervention group was emailed one peer and one expert (written by a team of behavioral scientists and clinicians) message weekly for 6 months. Compared with the control group, receiving a combination of peer- and expert-written messages significantly increased the odds of 7-day point prevalence cessation at 6 months (odds ratio [OR] 1.69, 95% CI 1.03-2.8) [12,13]. We further compared the effect of peer versus expert-written messages on intervention engagement, which was defined by return visits to the intervention website. Peer-written messages were more likely to generate a return visit to the intervention website (OR 2.03, 95% CI 1.74-2.35) than expert-written messages [14]. There were significant interactions of the effect of the peer-written message with time from registration (*P*<.001). As the time interval from registration increased, the benefits of peer-written messages were accentuated over time [14]. Preserving engagement in digital interventions is challenging yet critical to intervention fidelity and subsequent cessation. These results highlighted the benefit of peer message interventions [15]. Further details of our prior evaluation demonstrating the feasibility and effectiveness of a peer message intervention in a general population of smokers has been published [12-14].

Research shows that African American communities may be influenced by unique sociocultural dimensions, such as interconnectedness, level of health socialization, and ecological and health care system factors [16]. Messages written by health experts may not incorporate these dimensions. Thus, we hypothesize that African American smokers may particularly benefit from peer messages (messages written by African American smokers) as these messages could reflect shared experiences and exemplify difficulties of quitting, skills, and strategies needed for smoking cessation [17]. African American participants have reported greater engagement and improved outcomes when they felt a strong sense of community or support from social networks [18,19]. Prior studies that have tested a peer-driven digital intervention among African American participants have reported improved satisfaction and outcomes [17,18,20]. The peer messages developed in our prior work were written by a population of smokers (mainly non-Hispanic White smokers) [12,13]. To adapt the prior intervention, we asked African American adults, who were current or former smokers, to write messages to their peers guided by various hypothetical scenarios. In this paper, we focused on the qualitative assessment of the peer-written messages and report on the cessation strategies that emerged from the messages written by African American current and former smokers. In the future, these peer messages will be tested in an adapted and tailored technology-assisted behavioral intervention to promote cessation in African American smokers.

## Methods

### Study Design

We conducted a formative qualitative study of motivational messages written by African American current or former smokers. Writers (n=41) responded to various hypothetical scenarios (Multimedia Appendix 1) via an online semistructured questionnaire. Details on participant recruitment, data collection procedures, and analysis of peer-written messages are provided below. This protocol was approved by the University of Massachusetts Medical School Institutional Review Board.

### Participant Recruitment

Participants were recruited online via ResearchMatch between April 2017 and November 2017. ResearchMatch is an online web-based recruitment database freely available to individuals interested in volunteering for research studies [19]. Researchers can search the database and filter the list of volunteers by demographic information, such as age, race, ethnicity, and tobacco use. Participants were eligible if they self-identified as African American, were current or former smokers, were 18 years or older, and were willing to write messages for peers (n=41). Participants received a US $50 Amazon gift card for their contribution in the study.

### Data Collection

We collected data on sociodemographic factors and smoking characteristics. Sociodemographic factors included age, gender, education, and ability to pay for medical care. The ability to pay for medical care measure was used as a proxy for socioeconomic status, providing insights into household resources and demands placed on participants by medical expenditures [21,22]. Smoking characteristics included readiness to quit, number of cigarettes smoked per day, past year quit attempts, past 30-day use of menthol cigarettes, level of nicotine dependence, and electronic cigarette (or e-cigarette) use. The readiness to quit measure was based on stages of change as described in the transtheoretical model of behavior change [23], and was captured by asking current and former smokers to indicate whether they (1) were not thinking about quitting, (2) were thinking about quitting, (3) had already quit, (4) had set a quit date, or (5) had quit today.

Current and former smokers were matched to hypothetical scenarios by age group (45 years or younger and older than 45 years) and gender (male and female). We requested that participants write motivational messages about quitting based on each scenario. We suggested they describe experiences that might be useful to other smokers before, on, and after a set quit date. We also asked them to include suggestions for maintaining motivation and addressing barriers to quitting (eg, how to handle urges and cravings). Each participant responded to the following two hypothetical scenarios: for a smoker ready to quit and one not ready to quit. All responses to the scenarios were captured through a semistructured questionnaire that was distributed through Research Electronic Data Capture (REDCap) (Multimedia Appendix 1).

### Data Analysis

The percentages and mean distributions of smoker characteristics were calculated. Informed by our previously published methods [14], we reviewed all messages to identify those with specific characteristics. First, the messages had to be directly quotable, include sufficient details, and be self-explanatory. Directly quotable messages with sufficient details were those that were written in sentence form and contained clear and detailed information targeted to the scenario. Second, the messages had to have behavioral content that reflected personal quitting experiences. The underlying assumption for this criterion is that personal experiences are considered credible and trustworthy and therefore can potentially motivate behavior change [24]. Messages were selected by research team members with relevant expertise in smoking cessation (CSN and RSS), health behavior change (CSN, AJM, JMF, RSS, and JHW), and qualitative analysis (JHW and AJM). Out of the 1200 messages written, 318 messages (27% of the original messages) met the criteria.

Examples of the messages selected are as follows:

She can speak with doctor and discuss methods of quitting, and what's available to help her. Try to reach out and see if there's a quit smoking program that either she can go to in person or she can call when she has urges.

Figure out what triggers her to smoke throughout the day have a plan. If you typically smoke after meals, you might want to have something planned after you eat to keep mind off it. Sometimes certain rooms of the house cause cravings if that's where you smoked. You might want to change the decor, try to reduce the smell of smoke, etc., to make the environment less triggering.

Examples of the messages excluded are as follows:

Secondhand smoking kills.

The cause and the effect.

Two coders (CSN and DM) reviewed selected messages in an open coding process until concepts became apparent, at which point a code was assigned. Labels and definitions of themes were based on words in the message text. This approach to developing sets of codes is a grounded theory approach to qualitative data analysis that limits researchers from erroneously “forcing” a preconceived result [25]. Data analysis was conducted using MAXQDA qualitative data analysis software developed and distributed by VERBI Software based in Berlin, Germany.

## Results

### Demographic and Smoking Characteristics

Among the participants, 60% (25/41) were female. Most of the participants had a college-level education (27% [11/41] were college graduates and 59% [34/41] had attended some college or technical school). The daily average number of cigarettes smoked was 8.5 (SD 5.7). More than half (23/41, 56%) of smokers were thinking about quitting, and 29% (12/41) had set a quit date. About half (21/41, 51%) of the participants reported a past year quit attempt. Most participants smoked within 30 minutes of waking up (22% [9/41] within 5 minutes and 51% [21/41] within 6-30 minutes). Moreover, 27% (11/41) of smokers had used e-cigarettes some days or every day in the past 30 days. Among smokers who had ever tried e-cigarettes, 59% (17/29) had used e-cigarettes to quit or cut down on smoking (Table 1).

**Table 1:** Characteristics of current and former smokers who participated in an online study (N=41).

Characteristic			Value, n (%) or mean (SD)
**Gender**			
	Female	25 (60%)	
	Male	16 (39%)	
**Age**			
	25-34 years	12 (29%)	
	35-44 years	11 (26%)	
	45-54 years	10 (24%)	
	55+ years	8 (19%)	
**Education**			
	High school graduate	6 (14%)	
	College graduate	11 (27%)	
	Some college or technical school	34 (59%)	
**How hard is it for you (and your family) to pay for medical care?**			
	Very hard	7 (17%)	
	Hard	6 (14%)	
	Somewhat hard	13 (32%)	
	Not very hard	15 (37%)	
Cigarettes per day, mean (SD)			8.5 (5.7)
**Readiness to quit**			
	I am not thinking about quitting	3 (7%)	
	I am thinking about quitting	23 (56%)	
	I have set a quit date	12 (29%)	
	I quit today	1 (3%)	
	I have already quit	2 (5%)	
**Stopped smoking one day or longer in the past 12 months**			
	Yes	21 (51%)	
	No	20 (49%)	
**Past 30-day menthol cigarette use**			
	Yes	35 (85%)	
	No	6 (15%)	
**How soon after you wake up do you smoke your first cigarette?**			
	Within 5 minutes	9 (22%)	
	6-30 minutes	21 (51%)	
	31-60 minutes	4 (10%)	
	After 60 minutes	7 (17%)	
**Have you used/ever participated in tobacco counseling before?**			
	Yes	7 (17%)	
	No	34 (83%)	
**E-cigarette use in the past 30 days**			
	Every day	1 (3%)	
	Some days	10 (24%)	
	Not at all	30 (73%)	
**Reasons for using e-cigarettes^a^**			
	To quit/cut down on smoking	17 (59%)	
	In places I am not allowed to smoke	5 (17%)	
	Trying it out/experiment with it	7 (24%)	

^a^Question asked to smokers who have ever used e-cigarettes at least once (n=31).

### Frequencies of Subthemes in Peer-Written Messages

Results are organized via the following themes: behavioral strategies, seeking help, improvements in quality of life, attitudes and expectations, and mindfulness/religious or spiritual practices. Under the *behavioral strategies* theme, distraction strategies were the most frequently suggested behavioral strategies (referenced 84 times in the 318 messages). Other behavioral subthemes included evidence-based treatments/cessation strategies (referenced 41 times), avoidance strategies (referenced 37 times), removal of smoking triggers (referenced 28 times), use of rewards (referenced 34 times), setting restrictions on one’s smoking and goal setting (each referenced 23 times), and use of reminders (referenced 17 times). In the *seeking help* theme, subthemes included seeking help or support from family/friends or close social network (referenced 56 times), former smokers (referenced 24 times), and health care professionals (referenced 22 times). Subthemes that emerged from the *improvements in quality of life* theme included concerns about the negative health effects of smoking (referenced 13 times), improving one’s health (referenced 22 times), and quality of life (referenced 21 times). Subthemes that emerged from the *attitude and expectations* theme included practicing positive self-talk (referenced 27 times), autonomy/independence from the smoking habit and financial cost of smoking (each referenced six times), and reducing second-hand exposure to others (referenced five times). Within the *mindfulness/religious or spiritual practices* theme, the subthemes included use of self-awareness techniques (referenced 36 times) and religious or spiritual practices to cope (referenced 13 times). Example messages for each subtheme are provided in Textbox 1 and Textbox 2.

**Themes of behavioral strategies and seeking help, and their subthemes derived from messages (N=318) written by African American smokers who participated in our online study.**

**Theme: Behavioral Strategies**

Subtheme 1: Use of distraction strategies–Behavioral coping strategies or finding healthy ways to pass time (n=84 [number of times the subtheme is referenced in the messages])

Message examples:

- Focus on something else like exercise or reading. Find a relaxing music playlist and turn it on during breaks or at other times when you would smoke.
- Buy sugar free gum, lollipops and a squish ball. When a craving hit take a slow deep breath and then use one of the items listed above to get through the craving.

Subtheme 2: Evidence-based treatments/cessation treatments–Alternatives to smoking and use of treatments (n=41)

Message examples:

- Take smoking cessation medication from the start to help ease into the process of quitting. Use medications to help with cravings, really plan your strategies for difficult situations.
- Purchase gum, mints, cinnamon sticks -- something to use instead of cigarettes. Nicotine patches, or medications, such as Chantix may be helpful.

Subtheme 3: Avoidance strategies–Avoiding places, people, and things that remind one of smoking (n=37)

Message examples:

- People find it helpful to avoid situations in which they might be tempted to smoke, especially in the days right after quitting.
- Stay away from people, places, and things that trigger her to smoke. Now is a time to find new acquaintances. And be honest if they are about to smoke. If they care about what you are attempting to do for self, they won't smoke in front of you. If not, walk away.

Subtheme 4: Use of rewards–Messages focusing on use of rewards (n=34)

Message examples:

- Focus on the reward as a diversion and something to look forward to.
- Remind herself constantly of her reasons for quitting, and put the money she saves aside and plan a vacation or treat for herself to celebrate 1 year of being cigarette free.

Subtheme 5: Removal of smoking triggers–Advice about removing anything that elicits an urge to smoke and/or is associated with the smoking habit. Done ahead of time, pre-emptively (n=28)

Message examples:

- Remove all cigarettes, ash trays, and lighters. Every single lighter needs to be removed even the lighters in all of your purses. Search all of your purses for lighters and matches and spare cigarettes. Get rid of them all.
- Deep clean her house. Wash all of her clothes, ‘cause smoke tends to get trapped in them if you smoke a lot. Remove all ashtrays & lighters. And e-arrange her furniture with a different set up.

Subtheme 6: Setting boundaries/restrictions around the smoking habit–Proactive ways to reduce smoking that limit engaging in smoking (n=23)

Message examples:

- Make your home and your vehicles smoke free zones.
- Travel with less cash or debit card if possible, throughout the day to assist you in not purchasing cigarettes. Maybe keep one cigarette on you during long days out of house.

Subtheme 7: Goal setting (planning)–Messages that focus on preparation to increase chances of success or setting up a plan (prepare for a quit date, prepare rewards/goals, or set a quit date) (n=23)

Message examples:

- You should think about how you will handle situations where you are tempted you will be tempted to smoke.
- Come up with distractions and plans for what to do when a craving hit ahead of time. These could include things like drinking mint tea if you smoked menthols, chewing gum, eating celery or pretzel sticks so your hands do the same movement as smoking. Also calling friends and keeping your hands busy can help.

Subtheme 8: Use of reminders–Remember motivation/reasons/goals around quitting (n=17)

Message examples:

- Post notes in her home that encourage and remind her of how much she can enjoy life without smoking.
- Keep a small, laminated card in his wallet that reminds him of why he quit and the benefits he is now enjoying.

**Theme: Seeking Help**

Subtheme 1: Seeking support from family/friends or individuals in social network–Seeking support from friends and family to help quit (n=56)

Message examples:

- Have a list of friends and family to call and talk with for emotional support and encouragement.
- Pull family and friends into the process. Allow them to help you, but also make it clear that they're there for support. It's up to you to define and communicate how you need to be supported. That support need may change over time. Just be clear what you are looking for and what you are not looking for. Example: accountability vs nagging you to death.

Subtheme 2: Seeking support from former smokers–Seeking support from former smokers or smokers trying to quit (n=24)

Message examples:

- I would align myself with other smokers who have gone through the struggle to quit, similar to AA or NA as a support base since they know what you are trying to accomplish and have already been there.
- Find alternate support means such as a group of former smokers or other friends who have previously quit smoking.

Subtheme 3: Seeking help or support from a health care professional–Advice to seek professional support in-person (from physicians) or online to help quit (n=22)

Message examples:

- Ask her physician for help. Talk with her doctor or find a quit line to see what options are available, such as patches or medications that might make it easier to handle cravings.
- Talk to her doctor about the benefits of no smoking. Also get a physical and the doctor can tell her how much quitting can actually benefits her.

**Themes of improvements in quality of life, attitudes and expectations, and mindfulness/religious or spiritual practices, and their subthemes derived from messages (N=318) written by African American smokers who participated in our online study.**

**Theme: Improvements in Quality of Life**

Subtheme 1: Concerns about the negative health effects of smoking–Conveying smoking health-related concerns (n=13 [number of times the subtheme is referenced in the messages])

Message examples:

- She could live longer and possibly overcome some of her health problems by not smoking.
- It does not actually decrease your anxiety, it does nothing to change the stress you are experiencing, the negative health effects are well documented and affect every part of your body.

Subtheme 2: Improving one’s health–Focusing on improving one’s own health as motivation to quit (n=22)

Message examples:

- You will feel better and your health will improve. You will breathe better. You have more energy through the day. You face will start to look smoother and brighter.
- Thinking about the positive outcome (improved health, relationships) is a great way to stay focused.

Subtheme 3: Improving one’s quality of life–Improving one’s own quality of life as motivation to quit (n=21)

Message examples:

- She can get in tune with her taste buds. Food tastes different once you have quit smoking.
- You smell a lot better and are not self-conscious when stepping into a new crowd. Also, first impression is everything. When going to a job interview, the first scent of you is smoke. That's unattractive and may cost you a great opportunity.

**Theme: Attitudes and Expectations**

Subtheme 1: Practice positive self-talk–Ways to develop/maintain a positive mindset (n=27)

Message examples:

- Quitting is difficult, take time to congratulate yourself on your success.
- Tell herself that she not weak she can do this. Hype yourself up by telling yourself you got this. You can do it.

Subtheme 2: Autonomy or independence from the smoking habit–Freedom to do what you want and when without being dependent on the smoking habit (n=7)

Message examples:

- Living life on your schedule, not on tobacco bothersome regime.
- Not having to inconvenience yourself to go outside to smoke or wait to smoke in nonsmoking areas, no longer smelling like smoke.

Subtheme 3: Quitting to spend time with family and friends–Highlight improving one’s own health to spend time with family as motivation to quit (n=6)

Message examples:

- If you quit smoking now, you may be able to spend time with your grandkids at a theme park instead of in a hospital.
- Things to me about quitting smoking was how much more quality time I would have with my family.

Subtheme 4: Financial cost of smoking–Messages focused on money spent on cigarettes (n=6)

Message examples:

- The money spent on cigarette is more than the cost of the medications used for smoking cessation. You are literally setting your hard-earned money on fire.
- I was tired of the amount of money that is literally going up in smoke.

Subtheme 5: Reducing second-hand exposure to others–Highlight on harm to family members due to smoking (second-hand smoke health concerns) as motivation to quit (n=5)

Message examples:

- Secondhand smoke has been proven to impact others. Your smoking can harm your family and friends.
- Imagine having lost a pet due to smoking. If your dog develops breathing issues and can no longer leave in the home, you are changing the life of your pet by your habit. It is unfair.

**Theme: Mindfulness/Religious or Spiritual Practices**

Subtheme 1: Use of self-awareness techniques–Self-awareness on the smoking habit and/or quitting progress (n=36)

Message examples:

- Keep a journal noting his progress, feeling and other thoughts.
- Sit down and start a daily journal to keep track of her feelings and experiences while she goes through her journey.

Subtheme 2: Use of religious or spiritual practices to cope–Advice referencing use of prayer and/or spiritual practices to cope with cravings/urges and other challenges of quitting (n=13)

Message examples:

- Please pray constantly for the help from God that you will need.
- When you wake-up and pray to God to give you strength to make it through the day.

## Discussion

### Principal Findings

Our approach to adapting a peer messaging intervention generated a rich set of messages that can be incorporated into text message cessation interventions tailored to support African American smokers. The peer-written messages aligned with evidence-based practices for supporting cessation and corresponded to the following five themes: behavioral strategies; seeking help; improvements in quality of life; attitudes and expectations; and mindfulness/religious or spiritual practices. The themes reflected in the messages described in this paper can be applied to smokers at all stages of behavioral change.

Within the distraction behavioral theme, use of evidence-based treatments/cessation treatment, and avoidance were the most frequently referenced subthemes. Distraction is often suggested as a way to prevent relapse for smokers [26]. Distraction techniques recommended by participants included physical activity and listening to music. Engaging in short bouts of physical activity is associated with a reduction in smoking cravings [27,28]. Listening to music helps participants manage emotional states and cravings that may act as cues to smoke [28]. Participants also suggested use of evidence-based treatment or cessation strategies such as nicotine replacement therapy. African American smokers reported less positive attitudes toward using cessation treatments and are less likely to use a Food and Drug Administration–approved cessation aid compared to White smokers [29]. Increasing the use of evidence-based treatment/cessation strategies among African American smokers is an important research priority in tobacco cessation [30]. Having a peer suggest use of cessation medications may increase the uptake of evidence-based strategies among African American smokers, and this needs further research. Avoiding situations that trigger smoking or peers who smoke is also an often-suggested strategy for smokers interested in quitting [31].

Participants frequently suggested seeking help from family and friends, and health professionals during cessation. Past studies indicated that social network support significantly contributes to cessation in African American individuals who smoke [32,33], and ways to increase this support for cessation need to be explored further [34]. Peers also suggested requesting help from health professionals. Brief interventions from health providers are shown to increase cessation among smokers [35]. However, African American smokers are less likely to receive these interventions [29,36]. Taking the initiative to request for help from a health professional may alleviate differences in receiving cessation advice from providers among African American smokers.

Improvements in the quality of life theme encompassed benefits of quitting and motivations to quit smoking. The most frequent subthemes were (1) improving one’s health and (2) quality of life. Given that the health effects of smoking and benefits of quitting are well-known, peers highlighting the health benefits of smoking may serve as reinforcement to African American smokers. Participants also highlighted several benefits of smoking to improve one’s quality of life, including eliminating the smell of cigarettes, breathing more easily, and having better taste, which are linked to one’s intentions to quit [34]. Smokers are more receptive to information that emphasizes the benefits of quitting [37]. The added benefit of having these messages written by peers is likely to enhance their impact on cessation.

Subthemes under the attitudes and expectations theme included practicing positive self-talk, autonomy/independence from the smoking habit, and the financial cost of smoking. Practicing positive self-talk has been associated with an increase in self-efficacy [38], and in turn, an increase in self-efficacy is associated with smoking cessation [39]. African American smokers are likely to benefit from practicing general self-regulatory activities, including self-talk, as these have been shown to result in smoking fewer cigarettes for up to 15 months [40]. Participants also urged peers to consider the financial costs of smoking as a motivation to quit. Smoking-related cost is an important motivator for cessation among smokers in lower socioeconomic groups [41,42].

Within the mindfulness/religion or spiritual practices theme, smokers were advised to use self-awareness techniques and religious or spiritual practices as coping mechanisms during cessation. In our past work in which we collected messages written mostly by non-Hispanic White smokers (32 out of 39 participants) [14], mindfulness, religious, and spiritual practices did not emerge from the peer-written messages. Although not exclusive to African American communities, use of spiritual and religious practices, as a coping response to health issues, is often adopted in this group [40], with positive and promising results in health outcomes [43]. Other health behavior change studies in African American populations have shown more positive outcomes if participants reported having strong ties within a religious congregation [41]. Exploring the use of religious practices for cessation is particularly important for African American smokers, since they are disproportionately impacted by tobacco-related health conditions. Participants also advised use of self-awareness techniques, which are shown to be effective for reducing the number of cigarettes smoked and for relapse maintenance [44,45]. The inclusion of self-awareness messages in cessation messaging interventions will be valuable.

To our knowledge, this is the first study to explore the content (and frequency of the content) of cessation messages developed by African American smokers. Peer-written messages developed in this study can be used to support cessation efforts among smokers motivated to quit, as well as among those less motivated. Smokers who are motivated to quit may experience withdrawal symptoms and smoking cravings during a quit attempt [39]. Messages that highlight distraction and avoidance strategies are therefore relevant to this group. Smokers who initiate quit attempts are vulnerable to declines in motivation, which can lead to resumption of smoking. Messages that highlight healthier lifestyle modifications (eg, engaging in physical activities), along with positive reinforcements, may be particularly helpful for maintaining motivation. For smokers currently not motivated to initiate a quit attempt, challenges, such as low self-efficacy and lack of support, reduce the chances of early quitting success [39]. Messages from peers that highlight ways to increase self-efficacy or ways to tap into sources of social support may be of particular benefit to this group.

This study had some limitations. Recruitment through ResearchMatch resulted in enrollment of highly motivated smokers. Therefore, the subthemes obtained from the peer-written messages may reflect behavioral strategies implemented by highly motivated smokers and could differ from strategies that might be implemented by smokers less motivated to quit. Our sample lacked diversity, as we enrolled a high proportion of female, older, and highly educated current and former smokers. Given that smokers with low socioeconomic status are the most likely population to have difficulty quitting [32] and that smoking is more prevalent in men than women [33], health behaviors observed in our sample could differ from those present in other populations. The meaning of messages may not have corresponded with the coders’ interpretations of the messages. We limited the effects of the researchers’ perceptions on the study findings by implementing coding protocols that preserved and used original words written by smokers.

### Conclusion

For populations experiencing tobacco-related disparities, a reduction in smoking rates may require development of tailored cessation interventions. In this formative work, we were successful in developing a rich set of peer messages written by African American current and former smokers that incorporated many evidence-based strategies and can be used to support both low- and highly-motivated African American smokers. In future research, we plan to assess the impact of these peer-written messages within a technology-assisted intervention for smoking cessation among African American smokers.

## Appendix

Multimedia Appendix 1 Hypothetical scenarios used in data collection of peer-written messages.

## Abbreviations

OR: odds ratio
