# Supplementary material for: Written Advice Given by African American Smokers to Their Peers: Qualitative Study of Motivational Messages
Source: JMIR Form Res. 2021 Apr 30;5(4):e21481. doi: 10.2196/21481 (PMC8128361; doi:10.2196/21481)
Supplement: Multimedia Appendix 1 [file formative_v5i4e21481_app1.docx]

**Multimedia Appendix 1.** Hypothetical scenarios used in data collection of peer-written messages.

| 1. Phillip, a young African American man who is ready to quit smoking. 2. Daphne is a young African American woman who is ready to quit smoking. 3. Henry is a middle-aged African American man who is ready to quit smoking. 4. Leslie is a middle-aged African American woman who is ready to quit smoking. 5. Brian is a young African American man who is not ready to quit smoking. 6. Nicole is a young African American woman who is not ready to quit smoking. 7. Dennis is a middle-aged African American man who is not ready to quit smoking. 8. Margaret is a middle-aged African American woman who is not ready to quit smoking. | |
| --- | --- |
| **Example scenario for Phillip, a young African American man who is ready to quit smoking.** | **Example scenario for Brian, a young African American man who is not ready to quit smoking.** |
| (Phillip), is planning to quit but has not set a quit date. Think about what you did to prepare to quit smoking. What would you say to as he is getting ready to quit?  When getting ready to quit smoking, one of the most important things (Phillip), can do is __________.  Many people think of reasons why they want to quit before to them actually quitting. What would you tell (Phillip) to encourage him to quit smoking?  For (Phillip), some good reasons for quit smoking are _______ and __________.  When tempting situations arise, it is often hard for people to continue in their quit attempt. Think about what (Phillip) can do to stay focused on quitting.  Thinking about ________ can help (Phillip) stay focused on quitting.  To help (Phillip) stay focused quitting, he can ________________.  Set a Quit Day  Our young African American man, (Phillip), is now ready to set a quit date. Quitting is easier for people who prepare before their quit day. Think about some of the things (Phillip) can do to get ready for his quit day.  Before his quit day, (Phillip) can __________________.  (Phillip) may find it helpful to ___________________.  Help of friends and family is important for people when they are quitting. Think about how your friends and family may have helped you when you quit.  (Phillip) can ask people around him to help him by_________________.  On the Quit Day  It is now 8 o’clock in the morning on (Phillip)’s quit day. What should he do? Items in your environment often serve as reminders to smoke and may make it more difficult to quit. When you quit smoking, what items did you find necessary to remove from your environment to keep you from smoking?  When (Phillip) is preparing to quit, he may find it helpful to remove _________________.  Quitting day is a big change for people. What are some of the things that (Phillip) can do on his quit day to help him quit.  On (Phillip)’s quit day, he can ___________.  Some people seek help in stopping smoking such as from doctors, quit-lines, or medication. What help would you recommend (Phillip) use on his quit day?  To help (Phillip) quit he could ___________. | Many people think of reasons why they want to quit before they actually quit. What are some of the things that may encourage (Brian) to quit smoking?  One reason (Brian) may want to quit smoking is ________. This may benefit his life by________________.  One immediate change (Brian) may notice from quitting smoking is ___________.  Thinking about ________ can help (Brian) stay focused on quitting.  To help (Brian) stay focused on quitting, he can ________________.  Smoking affects all aspects of a person’s life. It is important to think about the effects smoking has on you and those around you. What effects might (Brian) notice in his life?  Smoking may negatively impact (Brian)’s life by _______________________.  Smoking may negatively impact the people around (Brian) by __________________.  Quitting smoking is hard. Think about some of the difficulties you had when you tried to stop smoking. What challenges might Brain face in quitting?  Some of the problems (Brian) may have in quitting are ______________, ____________, and __________. To overcome these problems, he can ______________, _________, and ___________.  Family and friends can play a big role in quitting smoking. Think about things your family and friends did to help you stop smoking. Did they also do things that made it more difficult for you to stop smoking? How can (Brian) get his family and friends involved in his quit attempt?  (Brian) can ask his family and friends to help him quit smoking by _________________________________________  If (Brian)’s family and friends are making quitting smoking more difficult for him he can______________________________________  Since (Brian) has not decided to quit smoking it sounds like he might benefit from learning more about smoking and quitting smoking.  What are some things you would suggest (Brian) do to learn more? ___________________________________  Smoking can be a very expensive habit. Did you think about how much money you would save when you were quitting smoking?  What would you encourage (Brian) to think about regarding the cost of smoking? _________________________________________  It sounds like (Brian) is not quite ready to quit. Sometimes it is difficult to make that leap.  What made you quit?_________________________________ |

Scenarios were adapted from a prior study.[17] Each of the questions were duplicated under each of the eight scenarios.
